# Using remote sensing data to study anthropogenic land degradation in Khulna Division, Bangladesh for SDG indicator 15.3.1

**DOI:** 10.1016/j.heliyon.2024.e38363

**Published:** 2024-09-27

**Authors:** Ehsanul Bari, Md Arif Chowdhury, Md Ismail Hossain, Mohammad Mahfuzur Rahman

**Affiliations:** a Department of Environmental Science and Technology, Faculty of Applied Science and Technology, Jashore University of Science and Technology, Jashore, 7408, Bangladesh; b Department of Climate and Disaster Management, Faculty of Applied Science and Technology, Jashore University of Science and Technology, Jashore, 7408, Bangladesh

**Keywords:** Sustainable development goal 15.3.1, Remote sensing, Trends.Earth, LULC, Land degradation

## Abstract

The Sustainable Development Goals (SDGs) include a goal on land degradation: indicator 15.3.1 (proportion of degraded land over total land). It is not always easy to monitor the SDGs, and remote sensing could be an effective tool for monitoring several SDGs. This study assessed land degradation in Bangladesh's Khulna Division over the past two decades. The Trends.Earth toolset was used to assess land degradation during the baseline period (2001–2015) and the reporting period (2016–2020). Inputs include data from the United Nations Convention on Desertification, and outputs include three sub-indicators: land productivity, land cover change, and soil organic carbon (SOC) stocks. Over the past 20 years, the land use and land cover, land productivity, and SOC content of the study area have undergone substantial changes. A significant change was observed in croplands, water bodies, and built-up areas. Croplands have been converted into settlements and tree cover. Nonetheless, there is an increase in land productivity in the area (>64 %) accompanied by a small percentage of decreasing productivity (approximately 9 %). Accordingly, the SOC in major land areas (84.68 %) is stable with 66,475 tons of carbon lost from croplands. Overall, this area reveals substantial progress in SDG indicator 15.3.1 with a clear transformation of degraded land (from 10.38 % to 8.46 %) into stable land (32.09 %–64.01 %). Land degradation is mostly seen in Khulna, Bagerhat, Satkhira, Kushtia, and Jashore areas. Land covers change for urbanisation, developments, water logging, and salinity intrusion cause land degradation. Despite poor representation of the SOC and normalised difference vegetation index datasets in the waterlogged areas, the Trends.Earth-generated results are informative and stand alone. With the results of this study, policymakers may be able to develop more appropriate land management plans by better understanding the complex interconnections of land change processes.

## Highlights

• Analyzed RS-derived datasets in a country with limited data for SDG 15.3.1.
• Croplands, water bodies, and built-up areas changed significantly.
• SOC stock change, land productivity, and conversion were highest on croplands.
• Water logging, salinity intrusion, and land cover change cause land degradation.
• Reports clear progress in SDG 15.3.1 with degraded land becoming stable.

## Graphical abstract

**Figure:**
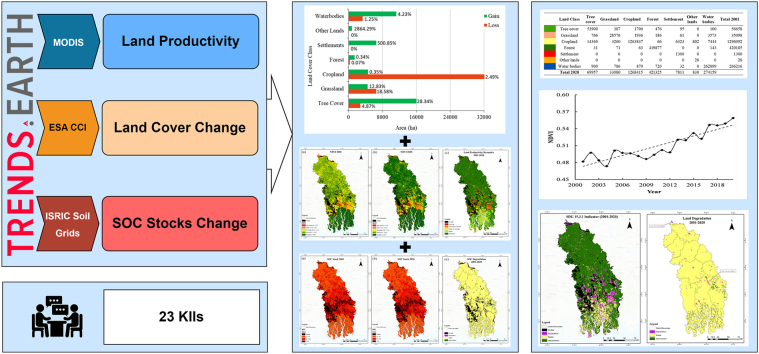

## 1 Introduction

The United Nations in 2015 has adopted 17 Sustainable Development Goals (SDGs) to be achieved by the year 2030. The goals encompass a wide range of issues, including poverty eradication, gender equality, clean energy, and climate action [1]. Tracking and reporting the progress of SDGs is crucial to assess the effectiveness of policies, identify areas that need attention, and ensure accountability in achieving the targets. A measurement framework comprising 232 indicators has been adopted by the United Nations Statistical Commission in order to monitor progress towards the SDGs and their 169 targets [2]. However, these indicators are being criticised for being difficult to measure, validate, and communicate due to their conciseness, subjectivity, and difficulty determining priorities and obtaining country-effective metrics [3,4]. It is therefore difficult to assess the overall progress of the SDGs, making it difficult to accurately measure the impact of initiatives [5]. Furthermore, the indicators are non-tailored to each country's context, which makes it challenging to accurately assess progress.

Bangladesh, a densely populated South Asian country, with its commitment to sustainable development, has made significant strides in various areas, yet it grapples with persistent challenges that demand innovative solutions. Bangladesh has made noteworthy advancements in several key SDG areas [6]. Poverty reduction efforts have led to a decline in the poverty rate, with a notable increase in access to education, gender equality and healthcare [7]. Additionally, progress in ensuring access to clean water, sanitation, and affordable energy has improved the overall quality of life for many Bangladeshis [8]. In the context of Bangladesh, several mechanisms are in place to monitor and report on SDG progress.

Bangladesh's approach to tracking and reporting SDG progress involves a multi-faceted strategy encompassing data collection, reporting mechanisms, international engagement, partnerships, and technological innovation. National Sustainable Development Strategy that outlines the country's approach to achieving the SDGs and serves as a guiding document, providing a roadmap for the implementation of sustainable development initiatives and policies [9]. Besides, Bangladesh has developed a comprehensive National Indicator Framework aligned with the global SDG indicators. This framework serves as the basis for tracking progress across all 17 goals and 169 targets [2]. It includes specific indicators, data sources, and monitoring mechanisms for each SDG, providing a structured approach to assessment. Bangladesh relies on a combination of official statistics, surveys, and administrative data.

While Bangladesh is actively tracking the SDGs, several challenges remain in data collection. It remains a significant challenge to ensure data quality and reliability. The accuracy of data may be limited in some cases due to out-of-date or incomplete statistical systems. Also, the country faces a number of challenges, including a lack of adequate human and financial resources for effective data collection, the lack of meaningful disaggregation in existing data collection systems, inadequate coordination among government departments and agencies responsible for collecting SDG-related data, gaps in incorporating technology into data collection processes, and an inability to access advanced data collection tools. Use of satellite-based Remote Sensing (RS) data could improve the situation.

By using RS satellite imagery, it is possible to easily, efficiently, accurately, and cost-effectively monitor several indicators associated with the SDGs [[10], [11], [12]]. RS offers accurate information on (i) land cover and land-use change (Goal 15: Life on Land) [13], (ii) sea level rise, temperature changes, and the extent of glaciers (Goal 13: Climate Action), (iii) real-time data on disaster events, aiding in early warning systems and facilitating timely response efforts (Goal 11: Sustainable Cities and Communities and Goal 13), (iv) water quality and availability (Goal 6: Clean Water and Sanitation and Goal 14: Life Below Water) [14], (v) mapping and monitoring urban expansion, infrastructure development, and changes in land-use patterns (Goal 11) [15], (vi) agricultural practices and crop health (Goal 2: Zero Hunger), (vii) mapping and monitoring biodiversity-rich areas (Goal 15), (vii) transportation networks, including road and rail systems (Goal 9: Industry, Innovation, and Infrastructure), (ix) suitable areas for renewable energy projects (Goal 7: Affordable and Clean Energy) and (x) air pollution levels (Goal 3: Good Health and Well-being).

In several studies, RS techniques were used to report study results related to SDGs in Bangladesh. Among their study interests are: (i) monitoring forest cover [[16], [17], [18], [19], [20], [21]], (ii) wetlands [[22], [23], [24]], (iii) riverbank erosion [[25], [26], [27], [28], [29]]; (iv) landslides [[30], [31], [32], [33]]. The literature may not sufficiently document the advancements made towards achieving the associated SDGs and the effectiveness of using RS to monitor SDGs in Bangladesh. The purpose of this study is to evaluate the effectiveness of RS technologies, coupled with Trends.Earth (hereinafter referred to as T.E) data sets, for monitoring the state and dynamics of land degradation (SDG indicator 15.3.1: proportion of degraded land over total land area) in a sub-national scale (Khulna Division) for Bangladesh.

T.E tool has been extensively used by researchers to investigate the progress of the SDGs or land degradation neutrality around the globe, particularly in the data scarce areas: Canada [34], Morocco [35], Ireland [36], China [37], Tanzania [38], Russia [39], Switzerland [40], Namibia [41], and South Africa [42]. The outcome of this study can enhance the country's monitoring and reporting capabilities for SDG indicator 15.3.1. It provides valuable insight into the tendencies of land cover and land-use changes, hotspots of land degradation, and supports the broader agenda of achieving sustainable land management and environmental conservation.

## 2 Materials and methods

### 2.1 Study area

**Fig. 1:**
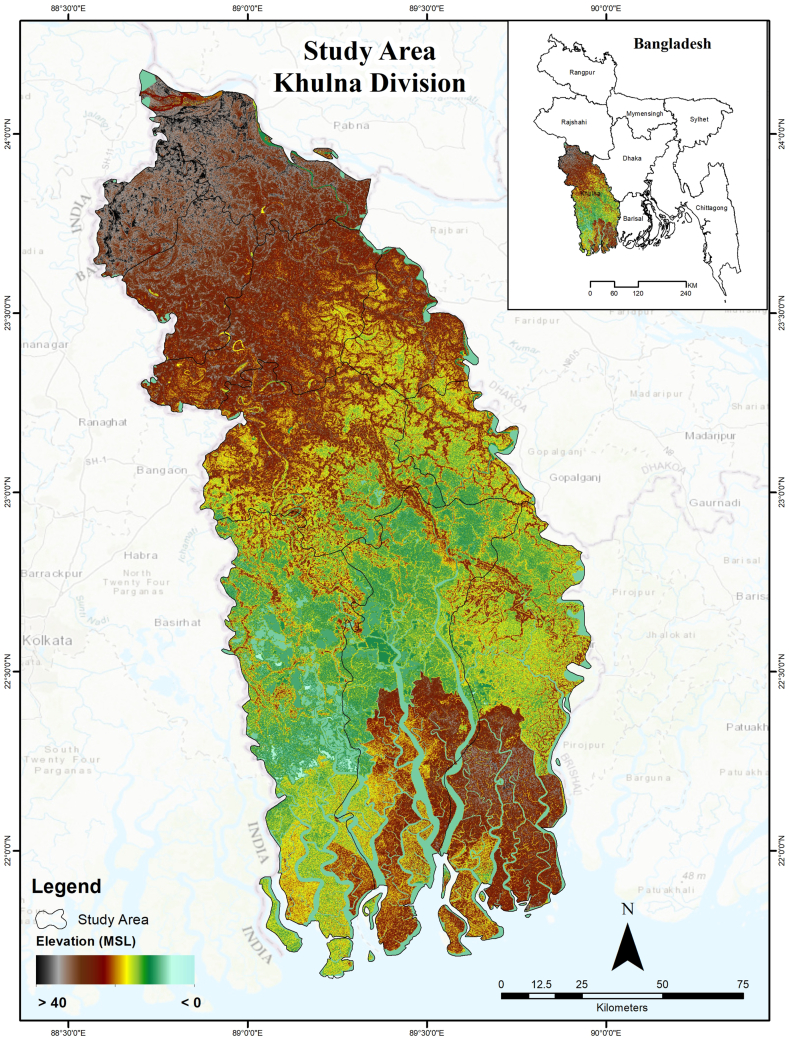
Study area map with elevation.

The Khulna Division, which is located in the southwest part of Bangladesh and consists of ten districts, is characterised by a tropical monsoon climate (Fig. 1). The study area covers an area of approximately 2.16 million hectares. Most of the land in the study area is used for agricultural purposes. Additionally, it has a variety of land uses, including mangrove forests, shrimp farms, land and seaports, coal-fired power plants, economic zones, and wetlands that are waterlogged. There are distinct dry and wet seasons in the region. In the study area, there is a diversity of land-uses, including agriculture, aquaculture, industries, and forests. Many factors are contributing to the degradation of the land in the area, including rapid urbanisation, the expansion of ports and export processing zones, the siltation of riverbeds resulting in waterlogging and salinity intrusion, and frequent natural disasters.

### 2.2 SDG indicator 15.3.1 calculation

With the help of the T.E tool (available online at: http://trends.earth), we have estimated the dynamics of land degradation in the study area between 2001 and 2020. We used 2001 to 2015 for the baseline, and 2016 to 2020 for the reporting period. T.E is a free and open-source tool that allows users to verify changes on the surface of the earth utilizing best available information from a variety of sources (details of the data source are provided in Table 1).

**Table 1:** Detailed characteristics of datasets.

Product Type	Dataset	Spatial resolution	Availability
NDVI	MOD13Q1	250 m	2001 - Present
Land Cover	ESA CCI Land Cover	300 m	1992–2020
Soil Organic Carbon	Soil Grids (ISRIC)	250 m	Static
Digital Elevation	SRTM V3	30 m	Static

Based on the United Nations Convention to Combat Desertification (UNCCD) proposed good practice guidance, T.E tool calculates SDG Indicator 15.3.1 using the UNCCD definition of land degradation. This tool uses datasets from UNCCD recommended repositories as inputs and produces information about land degradation by incorporating three sub-indicators, namely (i) land productivity, (ii) land cover change, and (iii) soil organic carbon stock, as outputs [43]. With the “One-Out-All-Out” (1OAO) principle, SDG indicator 15.3.1 estimation is done for every pixel based on land productivity, land cover, and SOC changes. According to 1OAO, if any of these sub-indicators show degradation, the overall SDG indicator 15.3.1 also shows degradation. If all the sub-indicators remain stable, the overall SDG indicator 15.3.1 shows stable. In cases where one or more sub-indicators are stable or improving without any degradation, the overall SDG indicator 15.3.1 shows improvement.

While adopting the T.E tool, we used datasets from 2001 to 2015 as the baseline period and estimated the change by comparing the status of the reporting period (2016 and beyond) [44]. In this study, the reporting period is 2016–2020. For more technical details about the T.E tool and its working principles, please visit https://docs.trends.earth/en/latest/for_users/features/landdegradation.html.

#### 2.2.1 Sub-indicator 1: Land productivity

T.E tool uses the Moderate Resolution Imaging Spectroradiometer (MODIS) datasets to estimate the Normalised Difference Vegetation Index (NDVI), which was then used to calculate land productivity dynamics. It calculates the ratio of the NDVI values of green (healthy) and red (stressed) reflectance from vegetation. Increasing or decreasing NDVI values throughout time serve as a measure of land productivity shifts. The trend in land productivity is provided in a logical five-category matrix (i.e., declining, early signs of decline, stable but stressed, stable, and increasing). Yearly mean NDVI scores for the respective years (2001–2020) were estimated from the MODIS dataset with the help of Google Earth Engine platform.

#### 2.2.2 Sub-indicator 2: Land covers change

To evaluate land cover changes, the T.E tool utilises the European Space Agency (ESA) Climate Change Initiative (CCI) land cover dataset [45]. The transition between land cover types is classified as improving, stable, or degrading. The ESA CCI dataset provides a yearly land cover classification in 37 global land cover classes at 300 m resolution for the period 1992–2020. Tree cover, Grassland, Cropland, Forest, Settlements, Other lands, and Water bodies are the seven aggregated land cover classes reported in this article, which are consistent than those of ESA CCI with minor modifications. We prefer to report “Forest” land cover instead of “Wetlands” due to the character of the land cover. The “Tree cover” land class denotes non-forest vegetation, while the “Forest” land cover denotes natural forest areas, specifically the mangrove forest of the study area. In order to compare the land cover changes between 2001 and 2020, a cross-tabulation matrix was used. Changes were classified as either positive or negative based on whether they led to a transition from one land cover category to another.

#### 2.2.3 Sub-indicator 3: Soil carbon stocks

As a default, the T.E toolbox uses the SOILGRIDS [46] dataset (https://www.soilgrids.org/) to calculate soil organic carbon stocks at depths ranging from 0 to 30 cm. The SOC is considered to be degraded if it decreases by more than 10 %. Using Eq. (1), the toolbox estimates the SOC.

(1) $${SOC}_{final}={SOC}_{ref}\times Land\;Use\;Coefficient$$

Where, ${SOC}_{ref}$ is the SOC in the baseline period. The *Land-use Coefficient* depends on the global climatic regions (e.g., Temperate Dry (f = 0.80), Temperate Moist (f = 0.69), Tropical Dry (f = 0.58), Tropical Moist (f = 0.48), and Tropical Montane (f = 0.64)).

### 2.3 Key informant interview

In order to obtain the opinion of experts on SDG indicator 15.3.1 indicator for the Khulna Division, twenty-three (23) Key Informant Interviews (KIIs) were conducted using a semi-structured open-ended questionnaire (Supplementary Part A). Respondents were those involved in issues related to land, environment, disasters, climate change, etc. Purposively, respondents were selected from government, non-government, and academic sectors. Supplementary Table 1 provides information regarding the respondents.

## 3 Results and discussions

### 3.1 Land cover dynamics

**Fig. 2:**
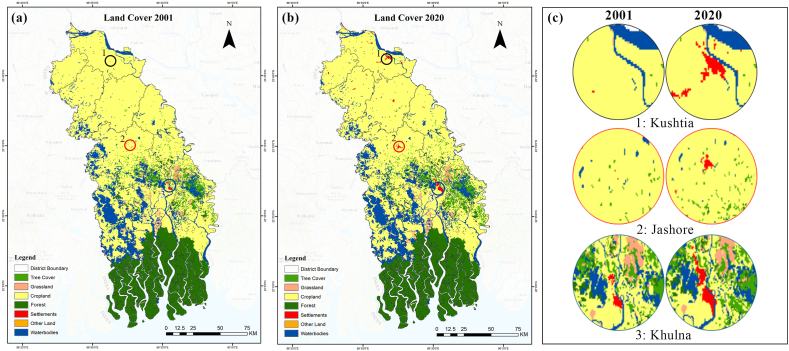
Land cover for the year (a) 2001, (b) 2020, (c) zoomed areas of selected hotspots.

The spatiotemporal distribution of land cover types observed in the study area is illustrated in Fig. 2(a)–(c). The area is dominated by agricultural land use (over 60 %), followed by world heritage mangrove vegetation, the Sundarbans (over 20 %), and water areas (approximately 13 %). Khulna, Bagerhat, and Satkhira regions have a higher percentage of water areas as a result of extensive aquaculture practices. Field experience, visual interpretation, and Google Earth-based validation indicate that T.E tool sets grossly underestimate settlements, homestead, and roadside tree coverage, perhaps due to coarse-resolution (300m) datasets. Nevertheless, it is estimated that settlements have expanded by 6 % in the past twenty years (see Table 2).

**Table 2:**
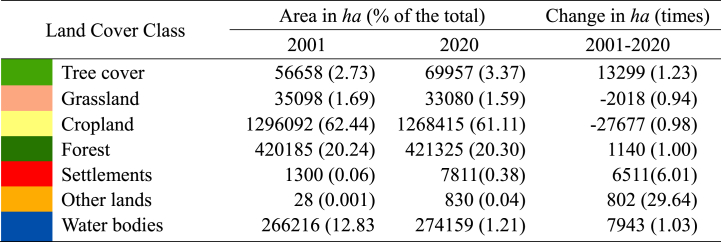
Changes in land cover classes from 2001 to 2020.

Based on Fig. 2(b), settlement area expansions are predominantly concentrated in and around three major districts (zoomed area) (see Fig. 2c). Besides, notable positive changes are observed in tree cover, forests, water areas and other land cover classes. Inversely, negative changes are evident in both cropland and grassland land cover classes. The dynamics of the land cover are consistent with Islam & Esraz-Ul-Zannat [47] and Hasan et al. [48]. Unplanned urbanisation, intensified agricultural practices, deforestation, and land cover degradation has been increasing in the Khulna Division [49,50]. Moreover, the increasing rate of climate extremes and salinity intrusion are also contributing to accelerating scenarios [48]. Local communities are mainly engaged in agriculture and fishing practices, which are highly interconnected with the changing patterns of land.

**Fig. 3:**
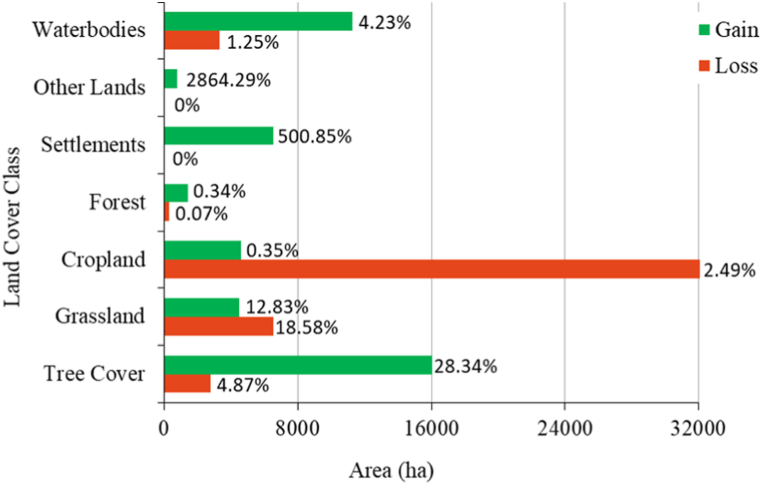
Gain and losses in all land cover types between 2001 and 2020, and % of area: (pixels changes for a class ÷ total area of the land cover map) × 100.

Table 3 illustrates land cover transition matrix during the study period (2001–2020). Land cover transition datasets indicate that croplands have been largely converted into tree cover, settlements, water bodies and grasslands. Other land cover types, such as bare areas, croplands that have been changed by sand filling, and sand deposits in rivers, exhibit the greatest gain after settlements. Fig. 3 shows the gain and loss of land cover types between 2001 and 2020. During the study period, the areas covered by trees and water bodies

**Table 3:**
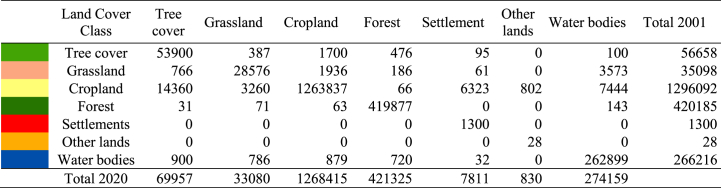
Land cover transition matrix during the study period (2001–2020).

experienced the most significant increase in terms of their respective areas. Croplands and grasslands are the primary inflows to water bodies. All land cover classes are experiencing new settlements.

**Fig. 4:**
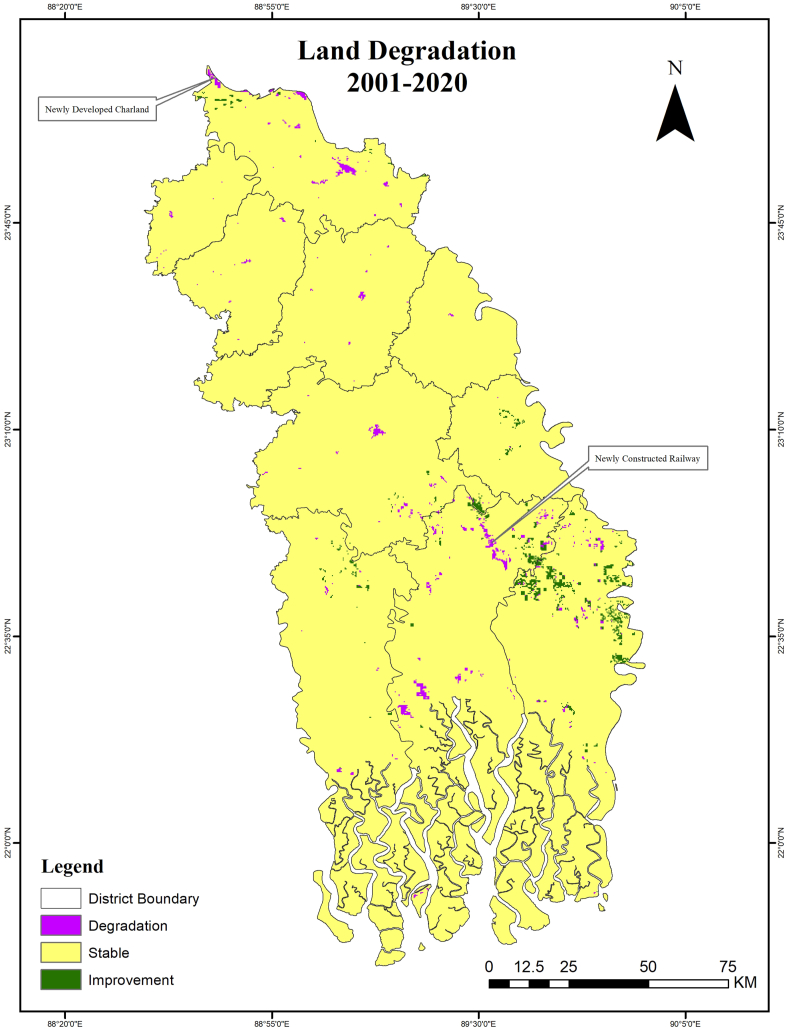
Status of land degradation during 2001–2020.

Based on land cover degradation criteria, 99.68 % of the studied region are stable between 2001 and 2020, as shown in Fig. 4. Additionally, there is a noticeable decrease in the areas of degraded land cover between the reporting years (0.12 %) and the baseline years (0.70 %), which is encouraging. Conversely, the improved land cover category reduced from 0.69 % (baseline years) to 0.20 % (reporting years).

The results of the KII, however, suggest a declining trend for tree cover, grasslands and forest areas in the study area. Urbanisation, shrimp farming, salinity intrusion, natural disasters, climate change, waterlogging, and industrialization are the dominant drivers of change identified by respondents. The slight expansion of the forest and other land categories could be attributed to the recent plantation of mangrove species in the Kochikhali area of the Sundarbans and the formation of new islands due to riverbed siltation respectively [51].

Respondents expressed mixed opinions regarding the trend of water areas. Although the majority of respondents believed that water bodies were diminishing, few argued for an increase. It is true that the area of water is expanding to some extent as a result of the establishment of numerous shrimp farms (locally referred to as “Gher”). On the flip side, people often convert the water areas (mostly road-side ditches or abandoned ponds) through sand filling driven by the economic (sand-filled and raised land's market price is substantially higher) or housing needs (Source: KIIs).

### 3.2 Land productivity dynamics

**Fig. 5:**
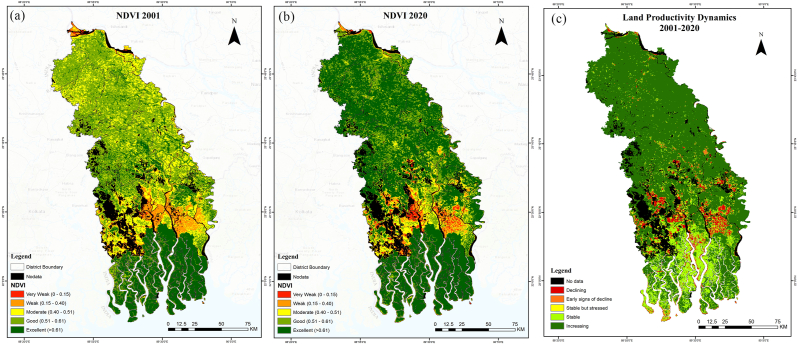
NDVI for the year (a) 2001, (b) 2020 and (c) productivity dynamics during 2001–2020.

Fig. 5 illustrates the spatial distribution of NDVI for the years 2001 (Fig. 5a) and 2020 (Fig. 5b). While the majority of the area displays healthy NDVI scores (>0.5), there are some patches of very low (0–0.15) and low (0.15–0.40) NDVIs. NDVI values were lower in the southern part of the study area, where water areas predominate.

**Fig. 6:**
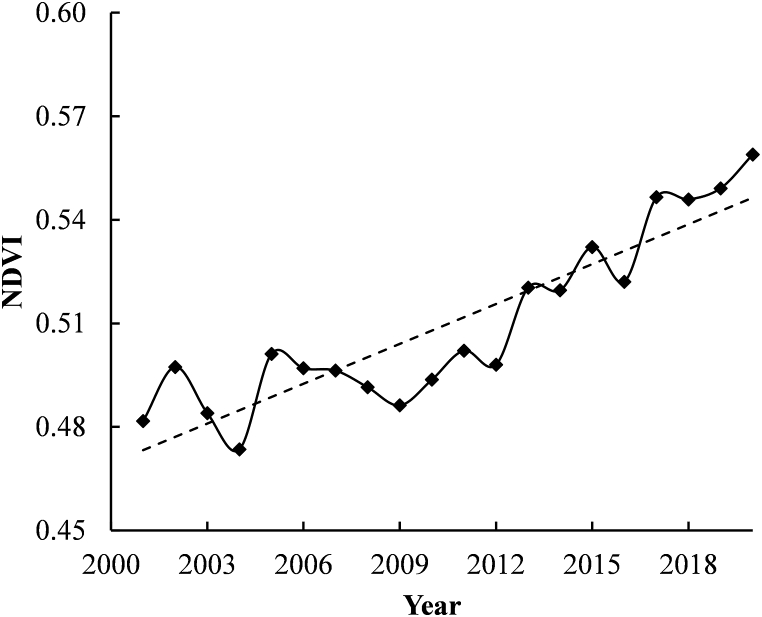
Mean annual NDVI trend between the years 2001 through 2020.

On the other hand, the yearly mean NDVI scores also demonstrate a positive trend over the past twenty years [52] with few inter-annual fluctuations (Fig. 6). A similar increase in NDVI was reported by Panday & Ghimire [53] between 1997 and 2006 in the Hindu Kush-Himalayan region, which they attributed to increasing crop intensities. According to BBS [54] and BBS [55], the Khulna Division has witnessed a rise in cropping intensities from 168 % to 207 % between 2012 and 2020. Consistent with the NDVI scores, it is evident that the area is experiencing increasing land productivity (>64 %). However, just a small percentage of the area shows indicators of declining productivity (approximately 9 %).

The increasing rate of disasters such as cyclones, storm surges, and floods hampers land productivity [56]. Fig. 5(c) indicates declining or early signs of decline hotspot areas are concentrated in the southern part of the study area. However, Mann-Kendall test on the productivity trajectory data suggests that the areas showing significant productivity increases (*p* < 0.01) reduced over the time period. Changes in land productivity data are presented in Table 4.

**Table 4:**
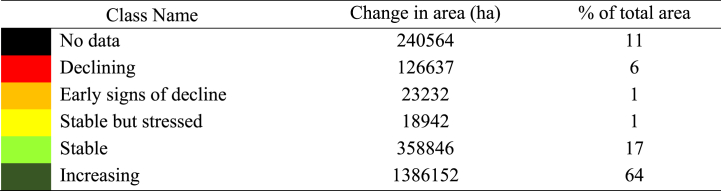
Land productivity dynamics from 2001 to 2020.

Experts and practitioners believe the trend in land productivity increment may be attributed to voluntary and guided changes in cropping pattern, cropping intensity, and agriculture extension activities. The Bangladesh Agricultural Development Corporation, the Department of Agriculture Extension, the Soil Resources Development Institute (SRDI), and the Bangladesh Agricultural Research Institute are all actively involved in supporting agricultural practices in the region. A minority of the key respondents argued that the output may be ascribed to the recent land reclamation from the waterlogged (i.e. Bhabadah) area.

### 3.3 Soil organic carbon

**Fig. 7:**
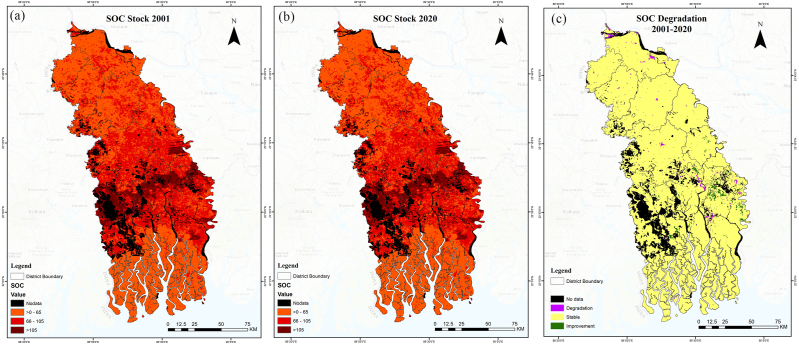
SOC stocks in (a) 2001; (b) 2020; and (c) change during 2001–2020.

As can be seen from the spatial distribution of SOC stock (Fig. 7), places that had waterlogging or were surrounded by water had the lowest concentrations as close to 0 tC/ha. The majority of these waterlogged and water-containing areas were located in the southern region of the study area. It should be mentioned that the SOC data from SOILGRID exhibits poor correspondence in the areas that are waterlogged and forested. As a result, we reported the SOC stock while masking out the water areas in Fig. 7.

On the other hand, areas associated with forests and croplands had moderate concentrations of SOC (0–65 tC/ha). Fig. 7 presents data on the SOC content specifically in the top 30 cm of soil. Nevertheless, Munny et al. [57] observed that the stock of SOC in croplands is greater in soils with greater depth. There is only a slight change in the concentration of SOC between the baseline period and the reporting period (Fig. 7(a) and (b)). Approximately 84.68 % of the land area under study experienced stable SOC, while 0.61 % experienced degradation. Fig. 7 (c) shows the degree of degradation of SOC between the baseline and the reporting years. As shown in Fig. 7 (c), most of the SOC has been degraded in and around city centres of Bagerhat, Khulna, Kushtia, and Jashore districts.

Over the course of twenty years, the soils in the study area (top 30 cm) lost approximately 66,475 tons of carbon. In comparison among the land cover classes, croplands had the largest decline in organic carbon content (Table 5), reflecting their agricultural and land management intensity [58]. On the other hand, settlement areas have shown the largest drop in SOC concentration, indicating the negative impacts of urbanisation. Several factors contribute to the loss of soil organic carbon, including salinity intrusion, excessive fertiliser application, shifting agricultural lands to aquaculture, natural disasters, and the increasing rate of human settlement.

**Table 5:** Changes in soil organic carbon stocks.

Land Cover Class	SOC (tC/ha)		SOC Stocks (’000 tC)		Change in SOC Stocks (’ 000 tC)
	Baseline period	Reporting period	Baseline period	Reporting period	2001–2020
Tree cover	89	91	5040	6335	1295
Grasslands	76	8	2674	2693	19
Croplands	72	72	93127	90899	−2229
Forest	53	53	22136	22244	107
Settlements	101	54	132	418	287
Other lands	16	17	4329	4683	354

### 3.4 SDG indicator 15.3.1

**Fig. 8:**
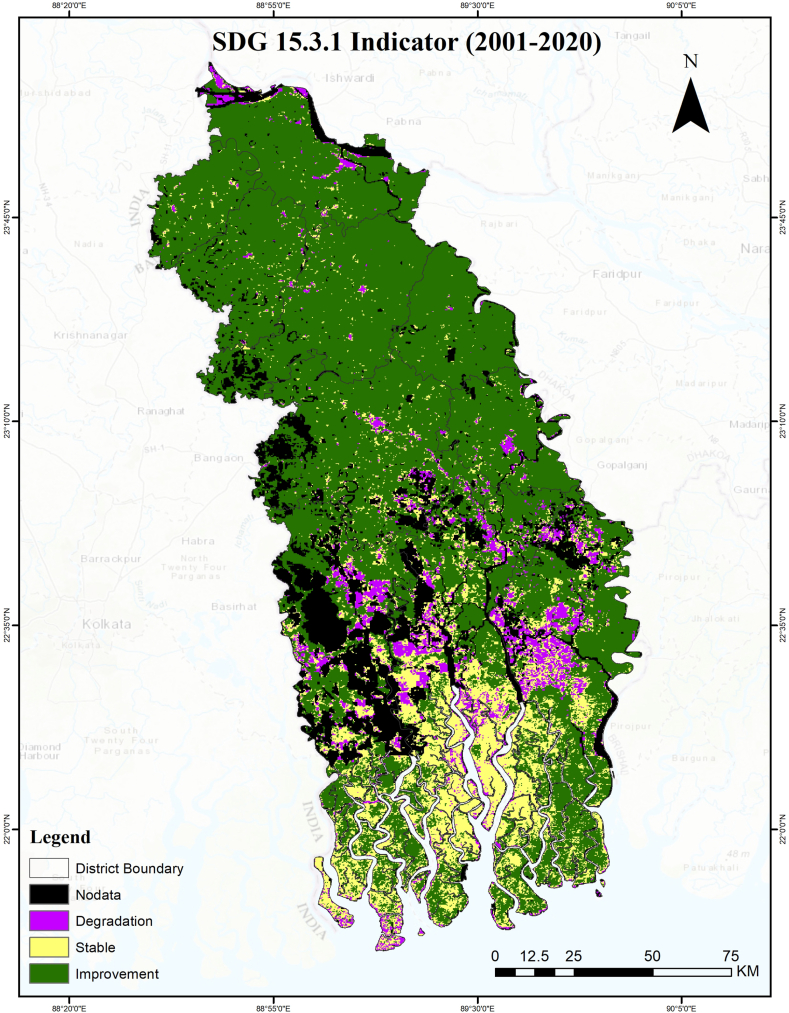
SDG indicator 15.3.1 aggregated map as of 2020.

Fig. 8 illustrates the status of aggregated SDG indicator 15.3.1, which measures the percentage of degraded land compared to the total land area. The assessment is based on the “one out all out” criteria applied to the study region. According to SDG indicator 15.3.1, the study region has a degraded land area of just 6.26 %, suggesting a progress in the SDG indicator 15.3.1. Table 6 shows that the reporting period (8.46 %) has experienced a decrease in land degradation compared to the baseline period (10.38 %). Conversely, there is a decline in the improving land class, with the percentage decreasing from 55.42 % in the baseline period to 25.41 % in the reporting period. The stable land class increased almost twofold in the reporting period (64.01 %) as compared with those of baseline period (32.09 %). The increase of stable land class indicates a transition of both the degradation class and the improving class into the stable class. The aggregated outcome, utilizing the baseline period and the reporting period, indicates a substantial enhancement in SDG indicator 15.3.1 within the study area.

**Table 6:**
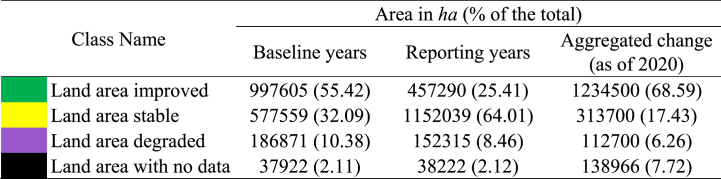
Changes in SDG indicator 15.3.1.

As a result of human activities like urban and agricultural development, fast-changing land cover provides an excellent indicator of changes in plant cover, ecological fragmentation, and land conversion due to human activities. The main causes of land degradation noted by the KIIs are changes in land use and cover, intensive farming, waterlogging, saline intrusion, riverbank erosion, and soil erosion on steeper slopes.

Land degradation is observed primarily in the Khulna, Bagerhat, Satkhira, Kushtia, and Jashore regions, whereas land degradation has improved in the eastern part of the Bagerhat District. Other than these five districts, five other districts have experienced minimal degradation between the baseline period and the reporting period. The recent establishment of a coal-fired power plant, together with its related development activities, has significantly contributed to the degradation of land in the Bagerhat District, making it a hotspot for land degradation. In addition, the intrusion of saltwater caused by shrimp farming or cyclonic flooding in the Bagerhat and Satkhira Districts might be significant factors contributing to land degradation. A significant portion of the Khulna and Satkhira Districts, which includes the Sundarbans mangrove forest areas, currently, exhibits a stable condition. However, if left unmanaged, this area has the potential to undergo degradation. Khulna and Satkhira Districts have significant concentrations of land degradation in the vicinity of waterlogged regions. During the Amphan cyclone in 2020, the embankment was damaged, which resulted in inundation of large areas of Pratapnagar, Satkhira and Koyra. By identifying hotspot areas for conservation of land from degradation, policymakers may be able to take necessary steps to prevent land degradation (Source: KIIs).

In terms of forest types, characteristics, and species distribution, as well as soil qualities, the Sundarban ecosystems have been altered and degraded, as KII has confirmed. Simultaneously, restoration initiatives have effectively mitigated the majority of the losses, which may have equalizing implications. Nevertheless, the productivity and SOC contents may not necessarily be significantly impacted by changes in forest composition on a shorter time scale. Consequently, the land degradation depicted in the current article may not accurately reflect the changes in forest, mangrove, and Sundarbans ecosystems.

It was confirmed by KIIs with the Department of Environment, the Forest Department, and the SRDI that the Bangladesh government had set six specific national land degradation neutrality targets, all of which were expected to be met by 2030. Improved soil fertility and carbon stock in 200 km^2^ of cropland are the first goal; land cover conversion in 600 km^2^ of forest area is the second; water logging is reduced in 600 km^2^ of area; soil erosion is reduced in hilly areas of 600 km^2^; non-saline land areas are protected from salinity intrusion in 1200 km^2^ of coastal zone area; and river bank erosion is reduced at a rate of 100 ha/year covering 100 km^2^ of area. These goals were decided upon at a consensus meeting with the technical committee, which is made up of stakeholders from several sectors and was established by the Ministry of Environment, Forests, and Climate Change. However, because of the overlapping interests and poor coordination within the sectors, reaching these aims will require overcoming difficult obstacles. In addition, data at the national level is limited in availability. The SRDI possesses an extensive collection of datasets in the subject of SOC. The SOC statistics produced by SRDI were estimated at various points in time and do not provide comprehensive country coverage on an annual or decadal basis. These datasets primarily focus on the agricultural land cover class and are not available in digital format. Furthermore, the Bangladesh Forest Department possesses the land cover maps of the country with at least 8–10 years gap between the updates (Source: KIIs). Under these circumstances, reporting the progress of SDG 15.3.1 using the country data could be extremely difficult. Meanwhile, T.E datasets could efficiently assist in downscaling the national targets into more precise regional or sectoral scales. Results from this study could aid in decision-making process for both national and local policymakers.

## 4 Conclusion

A number of international organisations are actively implementing the land degradation neutrality concept and the T.E calculation module as a means of providing a standardised method of monitoring and assessing SDG indicator 15.3.1 at the national and global levels. The T.E tool enables convenient surveillance of the state of global land degradation. T.E not only assists nations with data scarcity but also ensures consistency in reporting among the countries reporting to the UNCCD. Nevertheless, the regularities and the efficacy of the T.E produced results over a smaller (sub-national) geographic extent are not adequately comprehended. This study evaluated progress towards SDG indicator 15.3.1 on a sub-national scale based on the global datasets integrated with the T.E tool.

Over the past 20 years, the proportion of degraded land has decreased by 80.74 % in Khulna division. A simultaneous increase (200 %) in the stable proportion indicates a halt in the degradation process. Land degradation study results should provide policymakers with a template for enforcing SDG indicator 15.3.1 through restoration plans. In addition, other factors contribute to land degradation. Because of the complexity of this process, additional research is required into the socioeconomic and biophysical factors that lead to degradation. In Bangladesh, this is the first study on SDGs and land degradation, which may serve as a baseline for further studies on coastal divisions. As well, identifying hotspots of land degradation and the drivers of land use and land cover changes can help track SDGs and initiate future land-use policies. Monitoring the results of government activities is necessary to achieve land degradation neutrality and will help us comprehend the interconnectedness and dynamics of land transformation. Furthermore, it is imperative to generate country-specific datasets to monitor SDG indicator 15.3.1 more effectively and make informed decisions for the future. It is hoped that the findings of this study will guide policymakers to initiate appropriate and fruitful sectoral interventions throughout the Khulna Division. It is important to incorporate community-driven measures into current policies in order to address the changing patterns of land degradation from district to district. In the Khulna Division, Integrated Coastal Zone Management should be the prime focus to ensure sustainable coastal zone management [59]. With a proper plan and action, the national coastal zone policy should address issues related to land conservation directly. Moreover, Bangladesh Delta Action Plan incorporates the importance of an adaptive techno-economic plan that incorporates land-use and other environmental components [60].

## Data availability statement

Data will be made available on request.

## Funding

This study is partially funded by the Office of the Commissioner, Khulna Division (Grant No. #15; FY 2022-2023; Grant title: Ecosystem service value dynamics and the progress of SDGs against land use land cover scenarios of South-West Bangladesh).

## CRediT authorship contribution statement

**Ehsanul Bari:** Writing – review & editing, Writing – original draft, Visualization, Software, Methodology, Conceptualization. **Md Arif Chowdhury:** Writing – review & editing, Writing – original draft, Formal analysis. **Md Ismail Hossain:** Writing – review & editing, Data curation. **Mohammad Mahfuzur Rahman:** Writing – original draft, Supervision, Methodology, Conceptualization.

## Declaration of competing interest

The authors declare that they have no known competing financial interests or personal relationships that could have appeared to influence the work reported in this paper.
