# Supplementary material for: Using remote sensing data to study anthropogenic land degradation in Khulna Division, Bangladesh for SDG indicator 15.3.1
Source: Heliyon. 2024 Sep 27;10(19):e38363. doi: 10.1016/j.heliyon.2024.e38363 (PMC11471455; doi:10.1016/j.heliyon.2024.e38363)
Supplement: Multimedia component 1 [file mmc1.docx]

**Supplementary Part**

**Supplementary Part A: Questionnaire for KIIs**

**Using remote sensing data to study anthropogenic land degradation in Khulna Division, Bangladesh for SDG 15.3.1**

**Section 1: Personal Information**

| 1. | Name |  |
| --- | --- | --- |
| 2. | Age |  |
| 3. | Education level |  |
| 4. | Profession |  |
| 5. | Years of Working Experience |  |

**Section 2: Land Use**

| SI. | Questions | | Response (Put √ mark where applicable) | | |
| --- | --- | --- | --- | --- | --- |
|  |  |  | Increasing | Decreasing | No change |
| 1 | What is the changing pattern of land use (between 2001 and 2020) in the Khulna division for the different fields | Tree-covered areas |  |  |  |
|  |  | Grasslands |  |  |  |
|  |  | Forest |  |  |  |
|  |  | Settlements |  |  |  |
|  |  | Water bodies |  |  |  |
|  |  | Other lands |  |  |  |
| 2 | What are the major reasons behind the changing pattern of these land covers? (overall) |  | | | |

**Section 3: Land Productivity**

| SI. | Questions | Response (Put √ mark where applicable) | | |
| --- | --- | --- | --- | --- |
|  |  | Increasing | Decreasing | No change |
|  | What is the changing pattern of land productivity (in the years 2001 and 2020) in the Khulna division? |  |  |  |
|  | What are the major reasons behind that? (Overall) |  |  |  |

**Section 4: Soil Organic Carbon**

| SI. | Questions | | Response (Put √ mark where applicable) | | |
| --- | --- | --- | --- | --- | --- |
|  |  |  | Increasing | Decreasing | No change |
| 1 | Changing pattern of soil organic carbon (in the years 2001 and 2020) in the Khulna division | |  |  |  |
| 2 | What are the  changing  scenarios of soil  organic carbon  for the following  land covers (in  the years 2001  and 2020)? | Tree-covered areas |  |  |  |
|  |  | Grasslands |  |  |  |
|  |  | Forest |  |  |  |
|  |  | Settlements |  |  |  |
|  |  | Water bodies |  |  |  |
|  |  | Other lands |  |  |  |
| 3 | What are the major reasons behind that?  (Overall) |  | | | |

**Section 5: Degradation pattern of land cover**

| SI. | Questions | Response (Put √ mark where applicable) | | |
| --- | --- | --- | --- | --- |
|  |  | Increasing | Decreasing | No change |
| 1 | What is your opinion regarding the  degradation pattern of land cover? |  |  |  |
| 2 | What are the reasons or drivers behind the degradation/ improvement of land cover in  the Khulna division? |  |  |  |

**Section 6: Degradation pattern of land productivity**

| SI. | Questions | Response (Put √ mark where applicable) | | |
| --- | --- | --- | --- | --- |
|  |  | Increasing | Decreasing | No change |
| 1 | What is your opinion regarding the  degradation pattern of land productivity? |  |  |  |
| 2 | What are the reasons or drivers behind the degradation or improvement of land  productivity in the Khulna division? |  |  |  |

**Question:** What is your opinion on the involvement of GO/NGO/public representatives regarding the sustainable management of land uses in the Khulna division?

**Response:**

Supplementary Table 1: Background information of respondents

| **SI.** | **Gender** | **Age (years)** | **Education level** | **Profession** |
| --- | --- | --- | --- | --- |
| 1 | Male | 44 | Ph.D. | Professor, Khulna University |
| 2 | Male | 48 | Ph.D. | Professor, Jashore University of Science and Technology |
| 3 | Male | 43 | M.Sc. | Executive Engineer, Public Works Department |
| 4 | Female | 36 | M.Sc. | Associate Professor, Khulna University |
| 5 | Male | 32 | M.Sc. | Private sector |
| 6 | Female | 48 | M.Sc. | Project lead, Non-Government Organization |
| 7 | Male | 47 | M.Eng. | Senior Assistant Engineer, Local Government Engineering Department (LGED) |
| 8 | Female | 42 | MBA | Executive Engineer, Public Works Department |
| 9 | Male | 37 | M.Sc. | Project officer, Non-Government Organization |
| 10 | Male | 39 | M.Sc. | Architect, private sector |
| 11 | Male | 46 | Ph.D. | Government official, Social Wellfare |
| 12 | Male | 55 | MA | Senior executive, Non-Government Organization |
| 13 | Male | 33 | M.Sc. | Ph.D. student |
| 14 | Female | 45 | Ph.D. | Local stakeholder and UN official |
| 15 | Male | 43 | M.Sc. | Researcher, private sector |
| 16 | Male | 45 | M.Eng. | Government official, Bangladesh Agricultural Development Corporation |
| 17 | Male | 48 | Ph.D. | Professor, Jashore University of Science and Technology |
| 18 | Male | 40 | M.Sc. | Assistant Commissioner of Land, Jashore District |
| 19 | Male | 54 | M.Sc. | Government official, Department of Forest |
| 20 | Female | 41 | M.Sc. | Government official, Department of Environment |
| 21 | Male | 53 | M.Sc. | Government official, Soil Resources Development Institute |
| 22 | Male | 38 | Ph.D. | Professor, Jahangirnagar University |
| 23 | Male | 55 | Ph.D. | Government official, Department of Environment |
